# Full Digital Processing System of Photoelectric Encoder

**DOI:** 10.3390/s19224892

**Published:** 2019-11-09

**Authors:** Changhai Zhao, Qiuhua Wan, Lihui Liang, Ying Sun

**Affiliations:** Changchun Institute of Optics, Fine Mechanics and Physics, Chinese Academy of Sciences, Changchun 130033, China; wanqh@ciomp.ac.cn (Q.W.); lianglhciomp@126.com (L.L.); sun96ying@163.com (Y.S.)

**Keywords:** photoelectric encoder, full digital, error compensation, normalization

## Abstract

A photoelectric signal, output by a photoelectric receiver, may detrimentally change after the photoelectric encoder is used for a period of time or when the environment changes; this will directly affect the accuracy of the encoder and lead to fatal errors in the encoder. To maintain its high accuracy, we propose an encoder that can work in a variety of environments and that adopts full digital processing. A signal current that travels from the receiver of a photoelectric encoder is converted into a voltage signal via current limiting resistance. All signals are directly processed in the data processor component of the system. The encoder converts all the signals into its normalized counterpart. Then, the angle of the encoder is calculated using the normalized value. The calculated encoder angle compensates for any error. The final encoder angle is obtained, and the encoder angle is output accordingly. Experiments show that this method can greatly reduce the encoder’s volume. This method also reduces the encoder error from 167 arcseconds to 53 arcseconds. The encoder can still maintain a high accuracy during environmental changes, especially in harsh environments where there are higher accuracy requirements.

## 1. Introduction

A photoelectric encoder is a high-precision angle-measuring device that is widely used in various aviation, aerospace, and ground measurement systems [1,2,3]. When an external environment changes, or the encoder is used for a long period, the parameters of the light-emitting element and the receiving element (inside the photoelectric encoder) change [4,5,6,7], resulting in a deviation between the current output from the receiving element of the encoder and the ideal current, which affects the accuracy of the encoder [8,9,10]. When the deviation is large, the encoder error is so large that the encoder stops working. Any encoder error caused by environmental changes can be reduced, to a certain extent, by using complementary light-emitting and receiving elements at high and low temperatures [11,12]. The parameters of the light-emitting elements and the receiving elements cannot completely complement each other due to temperature changes; therefore, this method can only improve encoder errors caused by environmental changes but cannot completely eliminate encoder errors. By adjusting the resistance value of the digital potentiometer, the output amplitude of the encoder signal is within an ideal range. This method can also reduce the encoder errors caused by changes in the signal amplitude. However, this kind of processing circuit is complex and requires high-precision digital potentiometers. A processor can compensate for any longperiod of errors of the encoder and other parameters. Thus, its use conditions are limited.

Researchers have made several attempts to improve the resolution and accuracy of the encoder. For example, these have included using a high-precision image sensor to measure the encoder’s code plate position accurately [3,4], using multiple reading heads to improve the accuracy of the encoder [9], using the interference method to improve the quality of the moiré fringe signal, so as to improve the measurement accuracy [11], and using the contrast method to measure and ensure that the error of each position of the encoder is compensated for [13]. Previous studies have achieved good results. In this paper, on the basis of not increasing hardware or even reducing hardware, and instead only relying on algorithms to improve the measurement accuracy of the encoder, and when the output moiré fringe signal of the encoder changes due to the change of the external environment, the encoder can still maintain a high measurement accuracy.

In this paper, a digital processing system is used to process photoelectric encoder signals. The signal current from the encoder head’s receiver tube is converted into a voltage signal via current-limiting resistance. The signal voltage is input into the microprocessor for AD (Analog to Digital) conversion. The original signal from the encoder head is divided into a coarse and fine signal. When the encoder rotates, the microprocessor collects coarse and fine signals from the encoders in realtime; then, the microprocessor calculates the amplitude parameters of each coarse code signal and the fine code as the normalized parameters of the signal. The normalized parameter values of each channel of the encoder signal are recorded in the flash memory of the microprocessor, which is not lost after power failure. The microprocessor collects the actual value of each signal from the encoder, and this value is multiplied by the normalized parameter of the signal to obtain the normalized signal value. The normalized signal value is the value obtained by removing any parametric changes caused by environmental changes.

A reverse precise signal is added to remove errors caused by the eccentricity of the code disc [14,15]. The original encoder signal is a sinusoidal wave signal whose phase difference is 90 degrees, which is recorded in J0, J90, J180, and J270 parameters. The phase of J0 is set to 0 degrees, the phase of J90 is set to 90 degrees, the phase of j180 is set to 180 degrees, and the phase of j270 is set to 270 degrees. The difference between J0 and J180 is a sinusoidal signal (SIN); the difference between J90 and J270 is a cosine signal (COS). The arctangent of SIN/COS is the precise subdivision angle of the encoder. The original signal from the encoder’s photoelectric receiver is a current signal, which cannot be directly input into the AD converter, and needs to be converted to a voltage signal prior to input. The traditional processing method is that each receiver is connected with a potentiometer in series. When the current signal is fixed, the voltage of each signal can be adjusted by adjusting the resistance value of the potentiometer. Then, two signals with a phase difference of 180 degrees are sent to both ends of the analog amplifier. For example, J0 is sent to the positive (+) amplifier pin, j180 is sent to the negative (−) amplifier pin, and the output signal of the amplifier is a sine function. By adjusting the resistance of potentiometer and the amplification of the amplifier, the amplitude value of SIN is just the input range of the AD converter, and the amplitude value of the same COS is also consistent with the input range of the AD converter. In this system, traditional amplifiers are removed. The calculation method is used to ensure that the amplitude of the SIN and COS signals are consistent. Normalized and coarse code signals are transformed into binary terms. Then, the coarse code is decoded by looking up the value in a table, since the coarse code is corrected by the fine code. Finally, the fine and coarse code components are combined to obtain the appropriate encoder angle.

The encoder error is mainly divided into long-period and subdivision-type errors [16]. To improve the accuracy of the encoder, error-correction methods are added to the encoder system. After installation and adjustment, our encoder is installed alongside a high-precision encoder and rotated. The subdivision error and long-period encoder error are automatically measured by the comparison method. Then, the subdivision and long-period error are converted into tabular form, i.e., an error data table. The encoder uses a subdivision error table to correct the subdivision angle, while the long-period error is added to correct the subdivision angle before the angle output. Compared to a traditional encoder circuit, the data-processing circuit of the encoder greatly reduces the size of the data-processing circuit, improving the accuracy of the encoder and greatly improving the adaptability of the encoder to the environment. This method can reduce the encoder’s dependence on installation and adjustment accuracy, which is suitable for mass-produced encoders.

## 2. Principle

### 2.1. Hardware Principle

**Figure 1:**
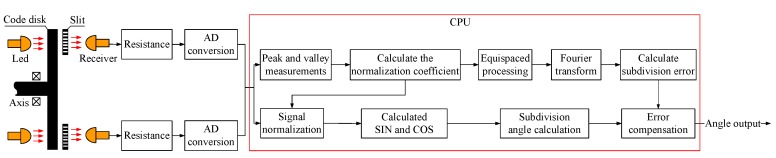
Schematic diagram of the encoder system.

The encoder system’s schematic diagram is shown in Figure 1. The encoder shaft drives the code disc to rotate. There are transparent and opaque patterns on the code disc. Different coders have different patterns. The light emitted by the light-emitting diode (LED) transmits the code disc, the slit, and is received by the receiver. The receiver converts the optical signal into a current signal. The resistance converts the current signal into a voltage signal and sends it to the AD conversion pin of the CPU for AD conversion. The converted AD value is converted into an angular value via an operation and output. There are 13 light-emitting elements and 13 receiving elements in the encoder. A total of 13 encoder signal currents are output from the receiving elements of the encoder, including nine coarse and four precise code signals. Then, the signal current is converted to a voltage signal through a current-limiting resistance, and the voltage signal is directly input into an AD conversion pin of the CPU for AD conversion. The size of the data-processing circuit is greatly reduced by eliminating conventional amplifiers and comparators. The CPU program is divided into two parts; one is the program for measuring and calculating the normalization coefficient and subdivision error. This part of the program does not have high requirements for real-time performance, nor does it have requirements for the length of calculation time, and it is executed in the main cycle. The other part is the program for calculating the output angle, which requires the encoder to output the current angle regularly, and has high requirements for real-time performance, so it needs to be placed in the execution of the interrupt. The data update rate of this encoder is 1kHz.

### 2.2. Software

Each coarse and fine encoder-coded signal is processed for AD conversion. Nine coarse signals need to be processed in binary form. Then, the angle of the encoder is calculated by coarse decoding. After adding four precise codes, the precise subdivision of the encoder is calculated.

Manufacturing and installation errors change the output signal amplitude of the encoder, and the result is a perturbed ideal signal amplitude. The signals are normalized to ensure that the signal amplitude is consistent with the ideal signal amplitude. The normalized amplitude values of all the fine code signals are ~±1024.That is, the peak value of all the signals after normalization is 1024, while the valley of all the signals is −1024 when the signal is centered at 0. When the encoder rotates, the minimum amplitude of the signal measured by the CPU is *A* and the maximum amplitude is *B*; thus, the amplitude of the signal is (*B* − *A*)/2, the signal is centered at (*B* + *A*)/2. In actual operation, if the voltage value of the signal at a certain position is measured as *x*, the normalized signal voltage value is measured as *y*, and their corresponding relationship is shown in Equation (1):

(1) $$y=(x-\frac{B+A}{2})\cdot \frac{1024}{\left(\frac{B-A}{2}\right)}$$

To prevent signal overflow, if the measured voltage value is greater than *B*, then we set the normalized voltage value directly equal to 1024. If the measured voltage value is less than *A*, then the normalized voltage value is directly equal to −1024, and the other values are calculated by Equation (1). Each precisely coded signal has a corresponding peak voltage *B* and valley voltage *A* (which is also called the amplitude normalization coefficient of the precise code signal).

The normalized J0 signal (denoted as J0’) and the normalized J180 signal (denoted as J180’) are differentiated to obtain a SIN signal. The normalized J90 signal (denoted as J90’) and the normalized J270 signal (denoted as J270’) are differentiated to obtain the *COS* signal. The resulting signal is equivalent to the two orthogonal signals (*SIN* and *COS*) formed by the fine code signal of the conventional encoder after being amplified by a differential amplifier.

The fine subdivision value of the encoder is calculated by Equation (2):

(2) $$\theta =\arctan(\frac{SIN}{COS})$$

θ is the fine code subdivision value.

The coarse signal of the small encoder generally has nine channels, which are recorded from *A*1 to *A*9. The coarse code signal is a square-wave signal. When the encoder rotates, the peak voltage of the signal is *B*1 and the valley voltage is *B*2. The central voltage of the signal is calculated by Equation (3):

(3) $$A=\frac{B1+B2}{2}$$

Since the coarse code signal is a binary signal, if the amplitude of the coarse code signal is greater than *A*, then the value of the coarse code signal after it is transformed into its binary form is 1; otherwise, the value is 0. *A* is the amplitude normalization coefficient of the coarse code signal. Therefore, each coarse code signal has its corresponding normalized amplitude coefficient.

During real-world operation, the encoder needs to determine whether it is being powered on for the first time. If so, the encoder is rotated for at least a week, and the normalized amplitude coefficient of each fine and coarse code signal is calculated and stored in the FLASH. Then, we ensure that the value can be read directly at the next start-up. If the encoder is not powered on for the first time, check whether the normalized coefficients of each fine code signal and coarse code information are stored in FLASH. Then, we determine whether the normalized coefficients are read directly. If the external environment has not changed, after viewing the stored normalization coefficient value in the FLASH, the stored normalization coefficient value can be directly read. If the encoder was not rotated for a week while the CPU processes the sample signals, the result will also get a peak-valley value. The peak-valley value obtained is not the final peak-valley value signal. If the encoder rotates for a complete period, the peak-valley value will be stored as the final peak-valley value signal.

When the external environment changes, the amplitude of the encoder’s fine code signal or coarse code signal changes. Then, the normalization coefficient value of each encoder signal may change. Therefore, when the encoder is working, the amplitude values of the encoder’s fine code signal and the coarse code signal are always recorded. After recording the stored normalization coefficient values in the FLASH, the encoder still calculates the normalized amplitude coefficients of each of the fine and coarse code signals in realtime. The normalized coefficient value, calculated in realtime, is compared to the stored normalized amplitude coefficient value to obtain a difference measure. If the difference is greater than the set threshold value, the normalized amplitude coefficient value, calculated in realtime, is read directly and replaces the original data during storage. If the difference is not greater than the threshold value, the stored normalized magnitude is read in order to prevent the angular value of the encoder output from being unstable due to frequent changes in the normalized coefficient value of the encoder.

### 2.3. Systematic Error Compensation

Due to errors in recording, adjusting, and manufacturing codes, even if all the coarse and fine signals collected by the encoder have an ideal signal amplitude, the final angular output value of the encoder will still have errors. Therefore, to further improve the accuracy of the encoder, it is necessary to compensate for the system encoder error. The systematic encoder error is mainly divided into long-period and short-period errors. A long-period error is the error of each coarse code of the encoder. A short-period error is the subdivision error of each fine-code period of the encoder, which needs to be compensated individually.

The long-period error data of the encoder are obtained by measurement. After the encoder is adjusted, the encoder needs to be tested while it is connected to the high-precision reference encoder. The encoder is rotated and compared with a high-precision encoder. The error of each coarse code of the detected encoder is obtained. In order to make the measurement more accurate, the error of the position where the fine code subdivision angle is 0 in each coarse code is taken during the measurement.

### 2.4. Fine Code Subdivision Error Compensation

Since there is a deviation between the Moiré fringe signal output by the photoelectric encoder and the ideal sine wave, there will be an error when the subdivision angle of the encoder is calculated by Equation (2). Especially when there are large harmonics in the signal, the subdivision error generated by the encoder will seriously affect the accuracy of the encoder, and the subdivision data needs to be compensated for.

Before subdivision error compensation, the fine code subdivision encoder error is first measured. Since the Moiré fringes have a smoothing effect on the signal, the difference of the fine code Moiré fringe signal in each period is small. For the convenience of calculation, it is considered that the fine code Moiré fringe signals of each cycle of the encoder are the same, and the fine code subdivision error of each signal cycle is the same.

The subdivision error of the photoelectric encoder can be divided into a DC (Direct Current) error, magnitude error, phase error, harmonic error, noise error, and quantization error. The actual moiré fringe signal collected by AD is a quasi sine wave signal, and the complete function expression of two channels of precise code signal is as follows:

(4) $$\left\{ \begin{array}{l} a=a_{0}+a_{m}•\sin(\theta +\varphi )+\sum_{i=2}^{\infty }a_{i}•\sin(i\theta +\varphi_{i})+\delta_{e}\\ b=b_{0}+b_{m}•\cos(\theta +\varphi_{b})+\sum_{i=2}^{\infty }b_{i}•\cos(i\theta +\varphi_{ib})+\delta_{e} \end{array}\right.$$

Each signal expression contains four subexpressions, of which *a*_0_ and *b*_0_ are the DC components, which are the source of the DC error; *a*_m_ and *b*_m_ are the magnitude of the fundamental signal, which are the source of the signal magnitude error; the phase difference of the two signals is the source of phase error; and $\sum_{i=2}^{\infty }a_{i}•\sin(i\theta +\varphi_{i})$ is the sum of the higher harmonic components and the source of harmonic error. $\delta_{e}$ is electrical noise, which is the source of noise error. Since the encoder angle data are represented by digital codes, a quantization error is generated. When the encoder stays at a certain position *θ*, the theoretical voltage value of the encoder moiré fringe output should be sin(*θ*) and cos(*θ*), but the actual output signal voltage is *u*_a_ and *u*_b_, as shown in Equation (4), which will produce the subdivision error.

The precision subdivision error of the encoder is expressed by the phase difference, which is the difference between the actual subdivision point phase arctan(ua/ub) and the theoretical subdivision point phase arctan(sinθ/cosθ). The fine code subdivision encoder error can be measured or calculated. Since the subdivision error obtained by measurement will have a large error, this paper uses the calculation method to obtain the subdivision encoder error. First, the encoder is rotated, and the Moiré fringe signal of a fine code period of the encoder is collected. The harmonic values of each encoder signal are obtained by Fourier analysis, and compared with the standard signal, and the subdivision error is calculated by Equation (4):

(5) $$d\theta =\phi -\theta =\arctan(\frac{u_{a}}{u_{b}})-\arctan(\frac{\sin\theta }{\cos\theta })$$

In Equation (4), sin*θ* and cos*θ* are the standard sine and cosine signals, and ua and ub are the two-way fine-coded Moiré signals of the actual encoder output, which contains various harmonics.

After an encoder’s subdivision error is calculated using Equation (4), the encoder’s angle can be compensated according to the value of the subdivision error. If the theoretical angle of point C at a certain angle is *θ*_C_, and the subdivision error of the position is *dθ_C_*, then the actual output value of the position is:

(6) $$\phi_{C}=d\theta_{C}+\theta_{C}$$

When the amplitude of a moiré fringe signal is acquired by a CPU via an analog-to-digital converter, given that the subdivision angle value of the position is $\phi_{C}$ by Equation (2), then the subdivision angle value of the position after compensation is $\theta_{C}$. In the actual program, for the convenience of the calculation, a table of subdivision compensations will be made. According to the subdivision value before compensation, the subdivision value after compensation can be output directly by looking up the value in a compensation table.

The influence of the product design, commissioning, and subdivision accuracy on the product is shown in Table 1. According to Table 1, most of the subdivision errors can be compensated for.

### 2.5. Error Synthesis

The calculated fine code subdivision encoder error is continuously changed, and the subdivision error of the start and end points of each cycle is 0. However, the encoder coarse code error obtained by measurement may be discontinuous. In order to make the angle information output by the encoder continuous, the coarse code encoder error needs to be smoothed. The linear smoothing method is used to smooth the error.

The start and end points of each subdivision cycle of the encoder correspond to a coarse code conversion point. If the coarse code error of the starting point of a fine code subdivision cycle is *E_a_*, the coarse code error of the end point of the subdivision cycle is *E_b_*, and if the length of the entire fine code is *C*, the coarse code error corresponding to the *D* point at the subdivision position is *E_D_*.

(7) $$E_{D}=(E_{b}-E_{a})\times \frac{D}{C}+E_{a}$$

The coarse and fine encoder error are added, which yields the final error of the position. After the signal acquisition command arrives, the CPU first collects the amplitude value of each encoder signal. Then, the position information of the encoder is determined. Then, the fine code subdivision error and the coarse code error of the position are found by looking up the table. The difference between the calculated position information and the error information, which is obtained from the position lookup table, is the compensated position information. The compensated position information of the encoder is output as the final angle information of the encoder.

## 3. Error Analysis

### 3.1. The Effect of Coarse Code Normalization on Accuracy

The coarse code encoder signal is used to determine which quadrant the fine code belongs to, which is equivalent to a large signal generated by the encoder. Since the coarse signal is necessary, the normalized coarse signal will not be affected by its binary value; so, the normalized coarse signal has no effect on the accuracy.

### 3.2. The Effect of Precision Code Normalization on Accuracy

When the encoder performs an AD conversion, there will be a resolution error, and the resolution error is one resolution of the AD. When the signal amplitude of the encoder coincides with the input range of the AD, the subdivision of the encoder is calculated according to Equation (2). The subdivision error caused by the insufficient resolution is the smallest. The smaller the signal amplitude of the encoder, the larger the resolution error.

**Figure 2:**
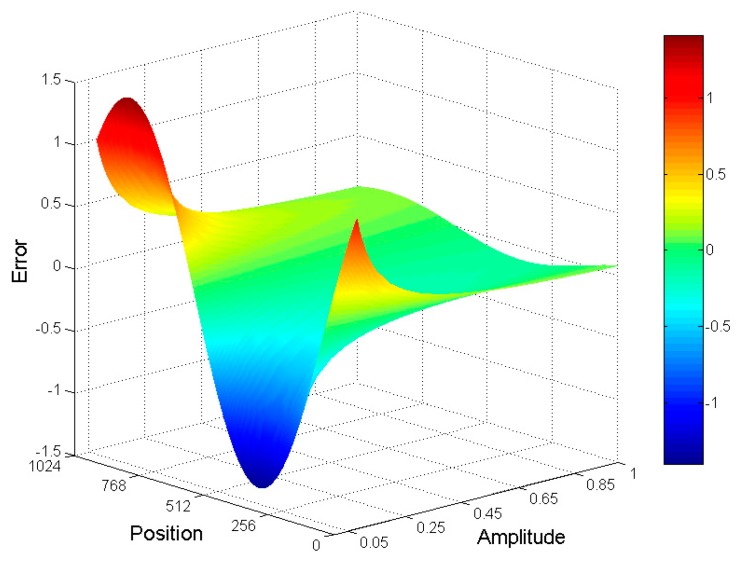
Subdivision error of the precision code signal when its amplitude is insufficient.

Generally, encoders are used when performing fine code subdivision. The number of fine code subdivisions is 1024, while the calculation error for less than 1 resolution bit is generally negligible. The resolution of the AD conversion used in this system is 12 bits. The resolution error caused by an insufficient signal amplitude of the encoder is shown in Figure 2. When the signal amplitude of the encoder is greater than 11% of the input range of AD, the calculation error yields an insufficient signal amplitude with a resolution that is less than 1, which can be neglected. In the actual encoder system, the signal amplitude of the encoder should be greater than 11% of the saturation amplitude.

**Figure 3:**
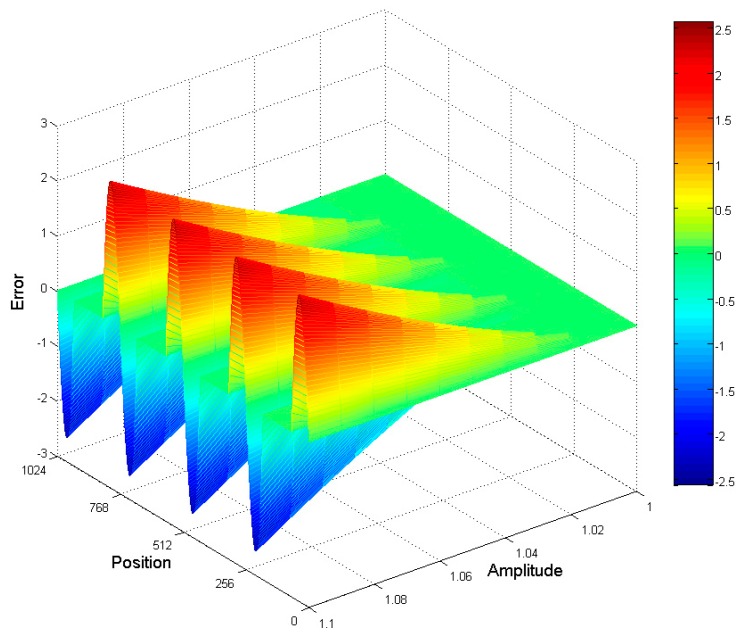
Subdivision error when the amplitude of the fine code signal is saturated.

When the signal amplitude of the encoder exceeds the range of AD conversion, the saturation error is introduced in the subdivision calculation of the encoder. The larger the signal saturation, the greater the error. The saturation calculation error introduced by the encoder’s signal amplitude saturation is shown in Figure 3. Thus, when the saturation of the encoder is less than 1.05 times the input range of the AD, the saturation error caused by the saturation of the signal’s amplitude is less than a single resolution term, which can be neglected. When using the encoder, try to avoid signal amplitude saturation.

### 3.3. Subdivision Calculation Error Introduced When the Speed of Encoder Varies

When calculating the subdivision encoder error, the signal harmonics of the encoder need to be determined. We need to sample the Moiré fringe encoder signal at equal intervals before calculating the signal harmonics of the encoder with a Fourier transform.

**Figure 4:**
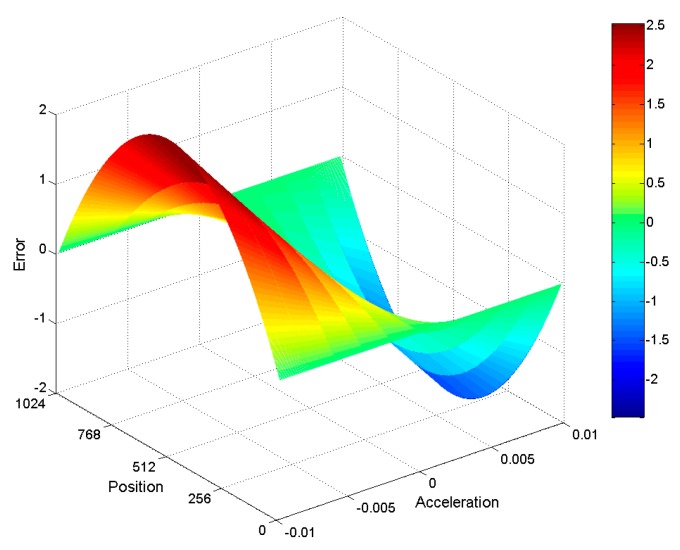
Subdivision error using unequal interval sampling.

Since the encoder data acquisition step uses fixed frequency sampling, the encoder needs to rotate at a constant speed during sampling. When the encoder rotates non-uniformly, the encoder subdivision error, determined by Equation (4), will be inaccurate. Figure 4 is the subdivision error caused by the encoder moving at a certain acceleration. When the relative acceleration of the encoder is less than 0.004, the maximum subdivision error caused by an uneven rotation of the encoder is a single resolution error, which can be ignored. See Figure 4 for more information. When calculating the subdivision error of the encoder’s signal sampling, the relative value of the speed variation of the encoder should be less than 0.004. Since it is difficult for the encoder to move uniformly when calculating the Moiré fringe subdivision error, three Moiré fringe signal periods need to be continuously collected. Additionally, information about the acceleration of the encoder needs to be recorded, particularly the time when the encoder rotates for each period of the Moiré fringe signal.

The non-isokinetic Moiré fringe signal motion is reduced to isokinetic motion according to the encoder’s accelerated motion. Then, signal harmonics are determined by applying a Fourier transform to the (Moiré fringe) signal of constant velocity motion. According to Equation (4), the subdivision encoder error is calculated as the value of the error compensation required to ensure a precise encoder signal.

When the encoder rotates, the data from the three complete fine code signal periods are continuously collected. We assume that the encoder rotates three signal periods (*T*_1_, *T*_2_, and *T*_3_) when *T*_1_ = *T*_2_ or *T*_2_ = *T*_3_. Thus, the encoder rotates at a uniform speed, and the collected data is equal-interval data, which can be directly transformed by a Fourier transform. If Equation (6) is satisfied, then the encoder’s motion is characterized by uniformly accelerated motion, which needs to be processed at equal intervals before the Fourier transform is applied. If these steps are not performed in order, then the data will not be processed.

(8) $$\frac{1}{T_{2}}-\frac{1}{T_{1}}=\frac{1}{T_{3}}-\frac{1}{T_{2}}$$

When the encoder moves with uniform acceleration, if the angle of each Moiré fringe period is *P*, then the initial speed and acceleration of the encoder are *V*_0_ and *a* such that:

(9) $$\begin{array}{l} P=V_{0}T_{1}+\frac{a}{2}T_{1}^{2}\\ P\times 2=V_{0}(T_{1}+T_{2})+\frac{a}{2}{\left(T_{1}+T_{2}\right)}^{2} \end{array}\}$$

To solve the system of equations:

(10) $$\begin{array}{l} V_{0}=\frac{P(T_{2}^{2}+2T_{1}T_{2}-T_{1}^{2})}{T_{1}T_{2}(T_{1}+T_{2})}\\ a=\frac{2P(T_{1}-T_{2})}{T_{1}T_{2}(T_{1}+T_{2})} \end{array}$$

the sampling time corresponding to the data points with equal intervals is calculated. The AD value of the sampling time consists of equivalent interval data, which can be used as a Fourier transform. When the calculated sampling time has no corresponding AD value, then the AD value of the current sampling time is obtained by a linear interpolation method via the AD value of the sampling time at the previous and the next moment.

## 4. Experiments

### 4.1. The Original Signal of an Encoder

**Figure 5:**
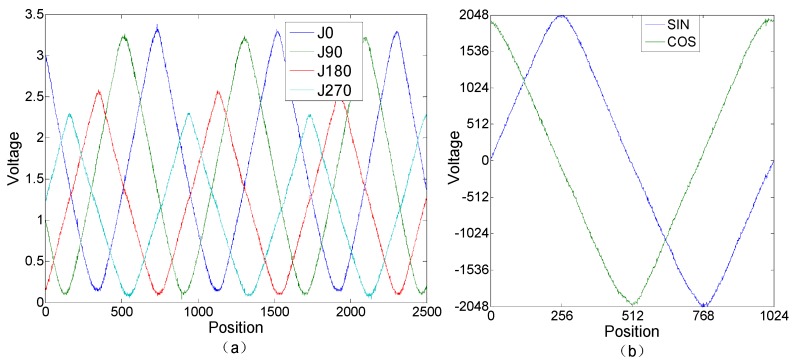
Encoder Moiré fringe original signals: (**a**) the four-way original Moiré fringe signals; (**b**) the normalized sinusoidal (SIN) and cosine (COS) signals.

A four-way original Moiré fringe signal of a photoelectric encoder is shown in Figure 5a. The encoder has a disc diameter of 36 mm and a resolution of 10 arcseconds. The signal of each Moiré fringe is normalized according to Equation (1). The difference between J0 and J180 (of a Moiré fringe signal) is used to acquire a SIN signal, while J90 and J270 are used to acquire a COS signal. Finally, the signal is processed at equal intervals to get a complete signal period. The normalized SIN and COS diagrams are shown in Figure 5b.

**Figure 6:**
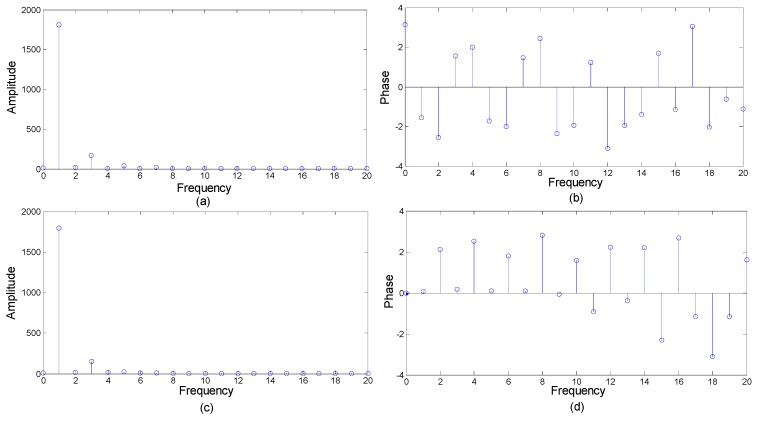
Amplitude-frequency characteristics of Moiré fringe signals: (**a**) the amplitude characteristics of the SIN signal; (**b**) the phase characteristics of the SIN signal; (**c**) the amplitude characteristics of the COS signal; (**d**) the phase characteristics of the COS signal.

A Fourier transform is applied to the normalized SIN and COS signals. The first 20 harmonic amplitude-frequency characteristics of the SIN signals are shown in Figure 6a,b. The first 20 harmonic amplitude-frequency characteristics of the COS signals are shown in Figure 6c,d.

**Figure 7:**
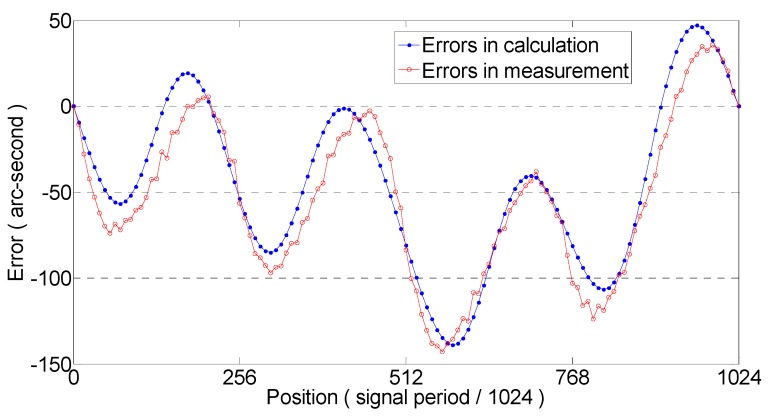
Subdivision error of encoder.

Figure 6 shows that the harmonics of the encoder moiré signal are mainly harmonics within five times, the signal amplitude of the higher harmonics is almost zero, and the influence is small. The higher harmonics of the Moiré encoder signal are noise components. Only the harmonics to the fifth order of the signal are used to determine the subdivision error. The calculated subdivision encoder error and the subdivision error obtained by the actual comparison test are shown in Figure 7. The subdivision error peaks and valleys obtained by the two methods are the same.

### 4.2. Error Compensation

**Figure 8:**
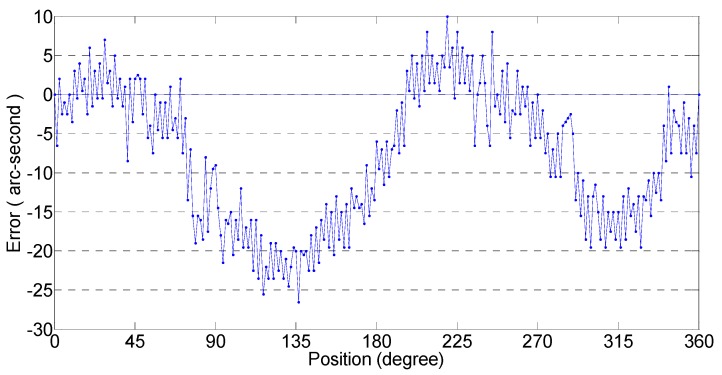
Encoder long-period error.

The reference encoder and the experimental encoder are coaxially connected. The resolution of the reference encoder is 0.15 arcseconds and the standard deviation is 1 arcsecond. The long period encoder error is measured using a comparison method, as shown in Figure 8. The long-period error of each subdivision point can be calculated by Equation (5) through combining the long-period error with subdivision error. Therefore, the comprehensive error of the position can be obtained, and the actual measured angle data can be compensated.

**Figure 9:**
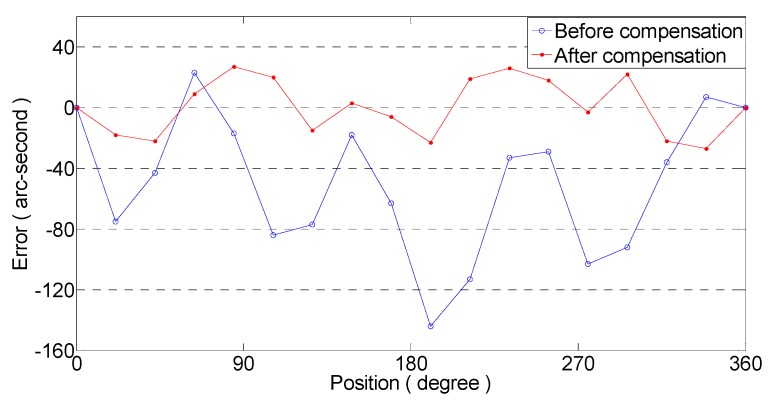
Errors of encoder before and after compensation.

The error curves of the actual test encoder, with and without compensation, are shown in Figure 9. The peak error of the uncompensated encoder is 167 arcseconds and the standard deviation is 46.48 arcseconds, while the peak error of the compensated encoder is 53 arcseconds and the standard deviation is 18.65 arcseconds. The processing circuit can greatly improve the accuracy of the encoder. When the external environment changes, the encoder can maintain highaccuracy.

## 5. Conclusions

When the external environment changes, the signal output of an encoder-receiving tube changes, which will cause a large error between the angular value of the encoder output and the actual angular value of the encoder. In this paper, an all-digital photoelectric encoder is studied. When the encoder works, the CPU collects the output signal of the receiver tube of the encoder in real time. After normalizing the signal, the angular value of the encoder is calculated, and the calculated angular value plus the error compensation value of the encoder is the final angular value of the encoder. The system encoder can effectively eliminate the error caused by the change in signal amplitude. Thus, the encoder can still maintain high accuracy when the environment changes, and greatly improve the environmental adaptability of the encoder.

## Figures and Tables

**Table 1:** The impact of design and debugging on subdivision error.

	DC Error	Magnitude Error	Phase Error	Low Harmonic Error	High Harmonic Error	Noise Error	Quantization Error
Code disc	■	●		●			
Axis	■	●	●				
Slit	●		■	■	○		
Optoelectronic devices	■	■	●		○	○	
AD			■		○	**□**	○
Power	■	●			○	**□**	
Temperature	■	●				**□**	
Manual adjustment	■	■	■				

■ The error is large and can be compensated; □  The error is large and cannot be compensated; ● The error is small and can be compensated; ○ The error is small and cannot be compensated.
